# Omentopexy Effect on Gastric Dilatation Post Laparoscopic Sleeve Gastrectomy: A Randomized Controlled Trial

**DOI:** 10.1007/s11695-025-08480-6

**Published:** 2026-01-27

**Authors:** Amir k. Abosayed, Mohamed Ahmed Farahat, Amr Mohammed Abd El Fattah Ayad, Arsany Talaat Saber Wassef, Ahmed Adel Shalaby Alattar, Shady Mohammed Tarek Gamal, Ahmed Yahia Abd EL Dayem

**Affiliations:** Cairo University, Giza, Egypt

**Keywords:** Laparoscopic sleeve gastrectomy, Omentopexy, Sleeve dilation, 3D gastric volumetry

## Abstract

**Background:**

Obesity is a global health crisis. Bariatric surgeries not only induce weight loss but also help in remission of these associated medical problems. Among the various bariatric procedures, laparoscopic sleeve gastrectomy (LSG) has become the most performed procedure worldwide. However, LSG has its own adverse effects. Suboptimal clinical response and recurrent weight gain are among the most challenging sequelae after surgery. Omentopexy is a novel step added to the conventional LSG. Our study aimed to assess the effect of omentopexy on gastric sleeve dilation following LSG and its impact on weight loss after surgery.

**Methods:**

This study included 100 patients who were recruited for LSG divided into 2 groups. Patients were randomly enrolled into the non-omentopexy group (underwent LSG without omentopexy) and the omentopexy group (underwent LSG with omentopexy). Gastric volumetry using 3D gastric computed tomography were done for all patients at Day 1, 6 month and 1 year after the operation.

**Results:**

There was no significant difference between the 2 groups at postoperative Day 1 (*p* = 0.12) regarding the sleeve volumetry. However, after 6 months and 12 months, the non-omentopexy group showed significantly greater volume increase (*p* < 0.001), there was no significant correlation between gastric sleeve volume and the EWL% and TWL% in Non-omentopexy and Omentopexy groups.

**Conclusion:**

Omentopexy step, although adds more to the operative time, but it may serve as a guard against postoperative sleeve dilation by stabilizing the staple line which can be a major role in maintaining sufficient weight loss and prevents recurrent weight gain on the long term.

## Key Points

LSG has become the most performed bariatric procedure worldwide.

Recurrent weight gain is among the most challenging sequelae after LSG.

Omentopexy fixes the sleeved stomach to the greater omentum.

Omentopexy may stabilize the staple line and prevent postoperative sleeve dilation.

## Introduction

Severe obesity is a growing global health crisis, with substantial social, economic, and medical consequences [1]. It is increasingly considered a pandemic, affecting quality of life and life expectancy across both developed and developing countries [2]. Bariatric surgery has emerged as the most effective long-term treatment for severe obesity, particularly in patients who do not achieve satisfactory outcomes with lifestyle or medical therapies [3].

Laparoscopic sleeve gastrectomy (LSG), first described in 2000 by Gagner as the initial stage of a biliopancreatic diversion with duodenal switch [4], has since evolved into the most commonly performed bariatric procedure worldwide. It provides effective and durable weight loss, with resolution or improvement of obesity-associated medical problems [5]. However, progressive dilation of the gastric remnant has been observed on follow-up. Whether this represents a normal physiological adaptation or a cause of recurrent weight gain and Suboptimal clinical response remains debated [6]. Importantly, failure after LSG is often associated with sleeve dilatation, leading some patients to require revisional surgery [7]. Although several studies have examined gastric volume changes after LSG, their findings are inconsistent, and the correlation between sleeve dilation and weight-loss failure remains unclear [8]. Some evidence suggests that sleeve dilation is a three-dimensional process, involving not only increased gastric capacity but also elongation of the staple line, with greater distensibility along the gastric curvature [9].

Omentopexy, a technique in which the gastric staple line is refixed to the greater omentum, has been proposed to stabilize the gastric tube and reduce postoperative complications such as reflux, food intolerance, and leaks [10, 11]. Despite its increasing use, the effect of omentopexy on long-term sleeve stability and gastric volume expansion has not been adequately investigated.

The aim of this prospective randomized controlled trial was to evaluate the impact of omentopexy on postoperative sleeve dilatation after LSG, using three-dimensional computed tomography (3D gastric CT) volumetry at 6 and 12 months postoperatively.

## Patients and Methods

This prospective randomized controlled trial was conducted at kasralainy hospitals between May 2023 and December 2024 and included 100 patients eligible for bariatric surgery. Inclusion criteria were adults with obesity I accompanied by obesity-related medical problems, deemed fit for surgery under general anesthesia. Patients who chose LSG after counseling, in accordance with departmental standards, were enrolled.

The study protocol was approved by the Institutional Research Ethics and written informed consent was obtained from all participants. Baseline assessment included detailed medical history, physical examination, and routine laboratory and radiological investigations. Preoperative esophagogastroduodenoscopy (EGD) was performed in all patients to exclude those with GERD or hiatal hernia. Eligible patients were then randomized into two study arms using a computer-generated allocation sequence.

### Surgical Technique

Patients in both groups underwent standardized preoperative preparation and general anesthesia. All procedures were performed with the patient in the supine position, legs apart, using a five-port technique following induction of pneumoperitoneum with a Veress needle at Palmer’s point.

The greater curvature was devascularized with an advanced bipolar sealing device (LigaSure), beginning 5 cm proximal to the pylorus and extending to the angle of His, exposing the left crus to ensure complete fundal mobilization. Posterior gastric adhesions were divided when present. A 36-Fr bougie was introduced trans-orally to the first part of the duodenum, and gastric resection was performed with a linear stapler (Endo GIA, Covidien).

In the non-omentopexy group, staple-line reinforcement was achieved with continuous oversewing: invagination with 2 − 0 PDS sutures up to the incisura angularis, followed by full-thickness continuous suturing of the remaining staple line. In the omentopexy group, the staple line was fixed to the mobilized greater omentum using 2 − 0 PDS sutures, starting 1 cm below the gastroesophageal junction and proceeding distally, with care to avoid injury to the gastroepiploic arcade. Seromuscular sutures with complete staple-line invagination were used above the incisura, while full-thickness sutures were applied below where gastric tissue was thicker.

Leak testing was performed intraoperatively with methylene blue. The resected stomach specimen was removed. Patients with intraoperatively detected hiatal hernia underwent concurrent repair and were excluded from the study.

### Postoperative Management

All patients were encouraged to ambulate early and were allowed oral fluids 2 h postoperatively, with subsequent diet advancement according to standardized written instructions. Prophylactic anticoagulation (enoxaparin 40 mg once daily) was administered for 2 weeks, and proton pump inhibitors (pantoprazole 40 mg once daily) were prescribed for 3 months.

On postoperative day 1, all patients underwent baseline gastric volumetry using three-dimensional computed tomography (3D-CT) with gas expansion. Patients ingested negative oral contrast (effervescent sodium bicarbonate) immediately prior to scanning to distend the gastric pouch. Scans were performed in the supine position on a multislice spiral CT system (GE, 128-slice), with acquisitions obtained during breath-hold and without intravenous contrast. Thin-slice images (1.5 mm) were reconstructed with a soft tissue kernel and transferred to a dedicated workstation. Volume-rendered images were generated, and sleeve volumes were automatically calculated by the software. All analyses were performed by a single abdominal radiology consultant with expertise in bariatric imaging.

Follow-up evaluations were conducted at 1, 3, 6, and 12 months postoperatively. Each visit included history taking, clinical examination, and assessment of BMI, %TWL, %EWL, complications, and associated medical problem remission. Repeat CT gastric volumetry was performed at 6 and 12 months using the same protocol. Patients who missed scheduled CT assessments were excluded from volumetric analysis.

### The Study Outcomes

The primary outcome was to evaluate the effect of omentopexy on gastric sleeve dilatation after LSG. Secondary outcomes included comparisons between groups in operative time, incidence of GERD, %EWL, %TWL, and other complications.

### Statistical Methods

The sample size was estimated using Statistics and Sample Size Pro, indicating 40 patients (20 per group) with a 20% dropout allowance. To strengthen study power and assess additional outcomes, 100 patients were recruited (50 per group).

Statistical analyses were performed using IBM SPSS Statistics, version 28. Categorical variables were presented as frequencies and percentages, and continuous variables as means ± standard deviations or medians with interquartile ranges. The chi-square test, Student’s t-test, and McNemar’s test were applied as appropriate. A two-sided *p* < 0.05 was considered statistically significant.

## Results

A total of 100 patients with obesity were randomized equally into the non-omentopexy group (*n* = 50) and the omentopexy group (*n* = 50).

### Baseline Characteristics

The two groups were comparable in age, sex distribution, BMI, and associated medical problems (Table 1).

**Table 1:** Baseline demographic and clinical characteristics

Variable	Non-omentopexy (*n* = 50)	Omentopexy (*n* = 50)	*p*-value
Age (years), mean ± SD	31.5 ± 5.5	33.1 ± 6.4	0.179
Weight (kg), mean ± SD	117.6 ± 2.8	122.3 ± 2.5	0.356
Height (m), mean ± SD	1.66 ± 0.06	1.68 ± 0.10	0.228
BMI (kg/m²), mean ± SD	42.5 ± 3.9	43.5 ± 5.4	0.288
Female sex, n (%)	41 (82%)	45 (90%)	0.248

No significant differences were observed

Preoperative laboratory values and imaging showed no significant differences. (Table 2).

**Table 2:** Preoperative laboratory and imaging findings

Variable	Non-omentopexy (*n* = 50)	Omentopexy (*n* = 50)	*p*-value
Hemoglobin (g/dL), mean ± SD	12.7 ± 1.2	12.9 ± 1.3	0.482
WBC (×10³/µL), mean ± SD	6.8 ± 1.4	7.0 ± 1.6	0.543
Platelets (×10³/µL), mean ± SD	251 ± 41	246 ± 44	0.621
Fasting glucose (mg/dL), mean ± SD	101 ± 12	104 ± 15	0.388
ALT (U/L), mean ± SD	26.1 ± 5.4	27.0 ± 6.2	0.459
AST (U/L), mean ± SD	24.8 ± 5.0	25.1 ± 5.7	0.719
Ultrasound: fatty liver, n (%)	18 (36%)	21 (42%)	0.541
EGD: hiatus hernia, n (%)	0	0	—

No significant differences in baseline laboratory or imaging parameters wereobserved

### Operative Outcomes and Early Complications

Mean operative time was significantly longer in the omentopexy group (94.7 ± 7.3 vs. 70.7 ± 5.8 min, *p* < 0.001). Postoperative complications were infrequent and did not differ significantly between groups (Table 3). No anastomotic leaks occurred in the omentopexy group, while one leak was reported in the non-omentopexy group. Median hospital stay was similar (2.3 vs. 2.0 days, *p* = 0.08).

**Table 3:** Operative outcomes and postoperative complications

Outcome	Non-omentopexy (*n* = 50)	Omentopexy (*n* = 50)	*p*-value
Operative time (min), mean ± SD	70.7 ± 5.8	94.7 ± 7.3	< 0.001
Any complication, n (%)	20 (40%)	11 (22%)	0.061
• Nausea	8 (16%)	4 (8%)	—
• Vomiting	4 (8%)	3 (6%)	—
• Fluid intolerance	5 (10%)	4 (8%)	—
• Leakage	1 (2%)	0 (0%)	—
• Bleeding	2 (4%)	0 (0%)	—

Operative time was significantly longer with omentopexy. Complication rates were comparable

### Gastroesophageal Reflux Disease (GERD)

At 6 months, new-onset GERD symptoms were reported in 14% of the non-omentopexy group and 8% of the omentopexy group (*p* = 0.34). At 12 months, persistent GERD symptoms were present in 71% of affected patients in the non-omentopexy group, whereas all patients in the omentopexy group achieved remission under PPI therapy (*p* < 0.001) (Table 4).

**Table 4:** New-onset GERD symptoms at 6 and 12 months

ssOutcome	Non-omentopexy (*n* = 50)	Omentopexy (*n* = 50)	*p*-value
6 months			
New-onset GERD symptoms, n (%)	7 (14%)	4 (8%)	0.337
12 months			
Persistent GERD, n (%)	5 (71% of affected)	0 (0%)	0.0004
Remission of GERD, n (%)	2 (29% of affected)	4 (100%)	0.0006

At 6 months, new-onset GERD symptoms were similar between groups. At 12 months, persistence of symptoms was significantly higher in the non-omentopexy group, while all patients in the omentopexy group achieved remission with PPI therapy.

### Weight Loss and Gastric Volume

Baseline gastric sleeve volumes were comparable on postoperative day 1. At 6 and 12 months, the non-omentopexy group demonstrated significantly greater sleeve dilatation compared with the omentopexy group (169.1 ± 26.1 vs. 145.5 ± 29.7 mL, *p* < 0.001; and 242.7 ± 36.4 vs. 180.2 ± 25.7 mL, *p* < 0.001, respectively).

Patients in the omentopexy group achieved superior weight reduction. At 12 months, mean weight was 65.9 ± 9.7 kg vs. 69.9 ± 5.2 kg (*p* = 0.032). %EWL was significantly higher in the omentopexy group at both 6 months (46.8 ± 9.0% vs. 42.3 ± 5.4%, *p* = 0.003) and 12 months (83.8 ± 12.2% vs. 74.1 ± 8.1%, *p* < 0.001). Similarly, %TWL was greater at 6 months (23.6 ± 3.9% vs. 20.0 ± 3.8%, *p* < 0.001) and 12 months (42.4 ± 5.4% vs. 40.3 ± 4.1%, *p* = 0.031) (Table 5). BMI reduction was comparable between groups.

**Table 5:** Weight loss and sleeve volume outcomes

Outcome	Non-omentopexy (*n* = 50)	Omentopexy (*n* = 50)	*p*-value
Sleeve volume (mL)			
Day 1	126.0 ± 21.6	134.2 ± 30.8	0.120
6 months	169.1 ± 26.1	145.5 ± 29.7	< 0.001
12 months	242.7 ± 36.4	180.2 ± 25.7	< 0.001
Weight (kg)			
6 months	94.1 ± 8.4	88.3 ± 7.1	0.003
12 months	69.9 ± 5.2	65.9 ± 9.7	0.032
%EWL			
6 months	42.3 ± 5.4	46.8 ± 9.0	0.003
12 months	74.1 ± 8.1	83.8 ± 12.2	< 0.001
%TWL			
6 months	20.0 ± 3.8	23.6 ± 3.9	< 0.001
12 months	40.3 ± 4.1	42.4 ± 5.4	0.031
BMI (kg/m²)			
6 months	34.0 ± 2.2	33.1 ± 4.2	0.192
12 months	25.4 ± 2.7	24.9 ± 3.1	0.388

Omentopexy was associated with significantly less sleeve dilatation and greater %EWL and %TWL at both follow-up points

### Correlation Analysis

No significant correlations were observed between sleeve volume and either %EWL or %TWL at 6 or 12 months in either group (Table 6).

**Table 6:** Correlation between sleeve volume and weight-loss outcomes

Variable	*r*-value	*p*-value
Non-omentopexy group (*n* = 50)		
Sleeve volume vs. %EWL (6 months)	0.12	0.38
Sleeve volume vs. %EWL (12 months)	0.15	0.29
Sleeve volume vs. %TWL (6 months)	0.10	0.42
Sleeve volume vs. %TWL (12 months)	0.14	0.33
Omentopexy group (*n* = 50)		
Sleeve volume vs. %EWL (6 months)	0.09	0.45
Sleeve volume vs. %EWL (12 months)	0.13	0.36
Sleeve volume vs. %TWL (6 months)	0.11	0.40
Sleeve volume vs. %TWL (12 months)	0.12	0.38

No significant correlations were observed between sleeve volume and weight-loss outcomes in either group

## Discussion

Laparoscopic sleeve gastrectomy (LSG) is currently the most commonly performed bariatric procedure worldwide [12]. Multiple studies have confirmed its efficacy in producing substantial weight loss, improving obesity-associated medical problems, and enhancing quality of life, with durable results extending into the long term [13]. Its primary mechanism is restriction of gastric volume, which limits food intake, making residual gastric capacity an important determinant of postoperative weight loss [14]. Typically, maximal weight loss is observed within the first 1–2 years, followed by stabilization and, in some patients, gradual recurrent weight gain, with mean long-term %EWL of 50–60% and BMI ranging from 30 to 35 kg/m² [15].

Progressive sleeve dilation has been reported after LSG, although it remains unclear whether this is a physiological process or a major contributor to recurrent weight gain [8, 16]. The phenomenon appears multidimensional, involving increases in both gastric length and width, with the greater curvature being more susceptible to expansion due to its flexibility [9].

Omentopexy has been proposed as an adjunct to stabilize the staple line and minimize postoperative complications, though techniques vary. Sharma et al. [17] used limited proximal and distal sutures, Pilone et al. [18] applied sealant with an omental flap, and Batman et al. [19] sutured the omentum to the staple line along its entire length. In our study, we performed continuous suturing of the omentum to the greater curvature to restore anatomical alignment and stabilize the posterior gastric wall.

Our findings demonstrated a significantly longer operative time in the omentopexy group, consistent with prior reports by Labib [20], Abou-Ashour [21], and Abosayed et al. [22], who described differences ranging from 5 to 25 min. Hospital stay was similar between groups, in line with Zarzycki et al.’s meta-analysis [10] and studies by Labib [20] and Afaneh et al. [23]. In contrast, Pilone et al. [18] and Sabry and Qassem [24] reported longer hospitalization in the non-omentopexy group.

Postoperative complications were uncommon. Although not statistically significant, bleeding and leakage were observed more frequently in the non-omentopexy group, supporting earlier reports that omentopexy may reduce these risks [17, 20, 22, 25]. With respect to GERD, de novo symptoms were comparable between groups at 6 months. However, at 12 months, persistent symptoms were significantly more frequent in the non-omentopexy group, while all omentopexy patients experienced remission under PPI therapy. Similar findings have been reported by Abosayed et al. [22], Abou-Ashour [21], and Labib [20], though others found no effect [25]. Omentopexy may mitigate reflux by stabilizing the gastric tube, correcting the His angle, and preventing twisting or kinking [26].

The primary endpoint, gastric sleeve volume, was comparable between groups on postoperative day 1. At 6 and 12 months, however, sleeve dilation was significantly lower in the omentopexy group. Comparable postoperative dilation after standard LSG has been reported by Braghetto et al. [8], Ali RF [27], Sabry et al. [28], and Vidal et al. [29], underscoring that sleeve expansion is common and progressive.

Regarding weight loss, both groups achieved comparable reductions in mean weight and BMI. However, the omentopexy group demonstrated significantly greater %EWL and %TWL at both 6 and 12 months. Importantly, no correlation was observed between sleeve volume and weight loss in either group, echoing the results of Ali RF [27] and Sabry et al. [28]. By contrast, Vidal et al. [29] and Pañella et al. [30] reported associations between gastric volume and weight loss, although these relationships weakened with longer follow-up, suggesting that additional factors such as baseline BMI may influence long-term outcomes.

To our knowledge, this is the first randomized controlled trial to directly compare sleeve volume changes after LSG with and without omentopexy. Our findings suggest that omentopexy attenuates postoperative sleeve dilation, decreases persistent GERD symptoms, and may enhance weight-loss outcomes.

## Strengths and Limitations

Strengths of this study include is one of a few prospective studies assessing the impact of omentopexy on Gastric Dilatation Post Laparoscopic.

Sleeve Gastrectomy in the literature, its randomized controlled design and the use of CT volumetry, which provides objective and reproducible assessment of sleeve capacity. Limitations include the single-center design, relatively small sample size, and limited follow-up duration, which restrict long-term generalizability.

## Conclusion

Although omentopexy increases operative time, it appears to reduce postoperative sleeve dilation and persistent GERD, while improving %EWL and %TWL at one year. These findings suggest a potential role for omentopexy in optimizing LSG outcomes. Larger multicenter studies with extended follow-up are warranted to confirm durability and long-term clinical benefit.

## Data Availability

Data accessibility exists.

## References

[1] Lee J. The obesity pandemic and the search for solutions. J Med Food. 2020;23(3):205–205. 32191578 10.1089/jmf.2020.29006.lee

[2] Hruby A, Hu FB. The epidemiology of obesity: a big picture. Pharmacoeconomics. 2015;33(7):673–689. 10.1007/s40273-014-0243-x PMC4859313 25471927

[3] Maciejewski ML, et al. Bariatric surgery and long-term durability of weight loss. JAMA Surg. 2016;151(11):1046–55. 27579793 10.1001/jamasurg.2016.2317 PMC5112115

[4] Varela JE, Nguyen NT. Laparoscopic sleeve gastrectomy leads the US utilization of bariatric surgery at academic medical centers. Surg Obes Relat Dis. 2015;11(5):987–90. 26003894 10.1016/j.soard.2015.02.008

[5] Gounder ST, et al. Costs of bariatric surgery in a randomised control trial (RCT) comparing Roux En Y gastric bypass vs sleeve gastrectomy in morbidly obese diabetic patients. New Z Med J (Online). 2016;129(1443):43. 27736851

[6] Noel P, et al. Revised sleeve gastrectomy: another option for weight loss failure after sleeve gastrectomy. Surg Endosc. 2014;28(4):1096–102. 24170068 10.1007/s00464-013-3277-9

[7] Barbiero G, et al. Relationship between gastric pouch and weight loss after laparoscopic sleeve gastrectomy. Surg Endosc. 2016;30(4):1559–63. 26150226 10.1007/s00464-015-4377-5

[8] Braghetto I, et al. Evaluation of the radiological gastric capacity and evolution of the BMI 2–3 years after sleeve gastrectomy. Obes Surg. 2009;19(9):1262–9. 19533260 10.1007/s11695-009-9874-y

[9] Baumann T, et al. Three-dimensional stomach analysis with computed tomography after laparoscopic sleeve gastrectomy: sleeve dilation and thoracic migration. Surg Endosc. 2011;25(7):2323–9. 21298527 10.1007/s00464-010-1558-0

[10] Zarzycki P, Kulawik J, Małczak P, Rubinkiewicz M, Wierdak M, Major P. Laparoscopic sleeve gastrectomy with omentopexy: is it really a promising method?—A systematic review with meta-analysis. Obes Surg. 2021;31(6):2709–16. 33677783 10.1007/s11695-021-05327-8 PMC8113139

[11] Abe Y, et al. 26th international Congress of the European association for endoscopic surgery (EAES), London, united Kingdom, 30 May–1 June 2018: video presentations. Surg Endosc. 2018;32:S361–429. 10.1007/s00464-018-6179-z 29675750

[12] Roux CW, Heneghan HM. Bariatric surgery for obesity. Med Clin. 2018:102(1):165–182. 10.1016/j.mcna.2017.08.011 29156184

[13] Angrisani L et al. IFSO worldwide survey 2016: primary, endoluminal, and revisional procedures. Obes Surg. 2018;28(12):3783–3794. 10.1007/s11695-018-3450-2 30121858

[14] Rosenthal RJ, Panel ISGE. International sleeve gastrectomy expert panel consensus statement: best practice guidelines based on experience of > 12,000 cases. Surg Obes Relat Dis. 2012;8(1):8–19. 22248433 10.1016/j.soard.2011.10.019

[15] Casella G, et al. Long-term results after laparoscopic sleeve gastrectomy in a large monocentric series. Surg Obes Relat Dis. 2016;12(4):757–62. 26806727 10.1016/j.soard.2015.09.028

[16] Lauti M, Kularatna M, Hill AG, MacCormick AD. Weight regain following sleeve gastrectomy—a systematic review. Obes Surg. 2016;26(6):1326–34. 27048439 10.1007/s11695-016-2152-x

[17] Sharma N, Chau WY. Remodifying omentopexy technique used with laparoscopic sleeve gastrectomy: does it change any outcomes? Obes Surg. 2020;30(4):1527–35. 31989384 10.1007/s11695-019-04357-7

[18] Pilone V, et al. Omentopexy with Glubran^®^ 2 for reducing complications after laparoscopic sleeve gastrectomy: results of a randomized controlled study. BMC Surg. 2019;19(Suppl 1):56. 31690312 10.1186/s12893-019-0507-7 PMC6829794

[19] Batman B, Altun H. Benefits of suture reinforcement in laparoscopic sleeve gastrectomy. Surg Laparoscopy Endoscopy Percutaneous Techniques. 2019;29(6):539–42. 10.1097/SLE.0000000000000722 31517747

[20] Labib MF. The omentopexy role in the prevention of post-operative gastric sleeve surgery complications. Egypt J Hosp Med. 2020;81(6):2199–204.

[21] Abou-Ashour HS. Impact of gastropexy/omentopexy on Gastrointestinal symptoms after laparoscopic sleeve gastrectomy. Obes Surg. 2022;32(3):729–36. 34870791 10.1007/s11695-021-05806-y PMC8866353

[22] Abosayed AK, Mostafa MS. Omentopexy effect on the upper Gastrointestinal symptoms and the Esophagogastroduodenoscopy findings in patients undergoing sleeve gastrectomy. Obes Surg. 2022;32(6):1864–71. 35320488 10.1007/s11695-022-05995-0 PMC9072512

[23] Afaneh C, Costa R, Pomp A, Dakin G. A prospective randomized controlled trial assessing the efficacy of omentopexy during laparoscopic sleeve gastrectomy in reducing postoperative Gastrointestinal symptoms. Surg Endosc. 2015;29(1):41–7. 24962864 10.1007/s00464-014-3651-2

[24] Sabry K, Qassem MG. The impact of routine omentopexy to staple line on the incidence of early postoperative complications after laparoscopic sleeve gastrectomy: is it worth? Egypt J Surg. 2018;37:(4):479–84.

[25] AlHaddad M, AlAtwan AA, AlKhadher T, AlJewaied A, Qadhi I, AlSabah SK. Omentopexy during laparoscopic sleeve gastrectomy: is it effective in reducing postoperative Gastrointestinal symptoms. A retrospective cohort study. Annals Med Surg. 2021;65:102369. 10.1016/j.amsu.2021.102369 PMC8121878 34026102

[26] Abdallah E, Emile SH, Elfeki H. Laparoscopic sleeve gastrectomy with or without staple line inversion and distal fixation to the transverse mesocolon: impact on early postoperative outcomes. Obes Surg. 2017;27(2):323–329. 10.1007/s11695-016-2277-y 27379770

[27] Ali RF, Tolba M, Ismail K, Ismail T, Lamey A, Balbaa MF. Volumetric pouch study after laparoscopic sleeve gastrectomy. Indian J Surg. 2024;86(Suppl 3):625–32.

[28] Sabry AA, Emara D. Volumetric pouch study after laparoscopic sleeve gastrectomy. Egypt J Surg. 2018;37:(2):265–9.

[29] Vidal P et al. Residual gastric volume estimated with a new radiological volumetric model: relationship with weight loss after laparoscopic sleeve gastrectomy. Obes Surg. 2014;24(3):359–363. 10.1007/s11695-013-1113-x 24242920

[30] Pañella C, et al. Correlation of gastric volume and weight loss 5 years following sleeve gastrectomy. Obes Surg. 2020;30(6):2199–205. 32065338 10.1007/s11695-020-04445-z

